# Vertical-X and stacked pulley stitches. Comment on “stacked stitch” for efficient closure of deep cutaneous defects”

**DOI:** 10.1016/j.jdin.2023.11.012

**Published:** 2024-01-06

**Authors:** Pedro Gil-Pallares, Alba Navarro-Bielsa

**Affiliations:** a Department of Dermatology, Miguel Servet University Hospital, Zaragoza, Spain; b Universidad de Santiago de Compostela, Santiago de Compostela, Spain

**Keywords:** buried suture, dead space, deep defect, deep wound, surgery, surgical dermatology

*To the Editor:* We have read with great interest the article by Glade et al^1^ in which they provided an interesting approach to the challenge of closing large surgical defects without dead space. Although this is an easy solution to perform, it reminds us of a kind of vertical purse-string suturing rather than 2 superimposed mattress stitches to ensure that a seroma or liquid collection could potentially form in the middle, especially in cases with high tension. To prevent this virtual space, we use a variant that crosses the surgical defect in the central area (Fig 1, *A*). The stitch starts in deep subcutaneous tissue and may include deeper tissues such as fascia or periosteum; further, it ascends and exits through superficial subcutaneous tissue or reticular dermis (step 1). It crosses to the contralateral side, enters at the same level, and ascends to the superficial or papillary dermis, where it exits (step 2). It crosses again, enters at the same level in the other wall of the surgical defect, and descends and exits at the superficial subcutaneous tissue or reticular dermis (step 3). It crosses and enters at the same level on the opposite side and descends to the deepest part of the defect (step 4), where it exits and is knotted with the other end. In this way, a vertical-X–shaped stitch is obtained, which brings both walls together at the lower, middle, and upper levels (Fig 1, *B*), giving the stitch its name.

**Fig 1:**
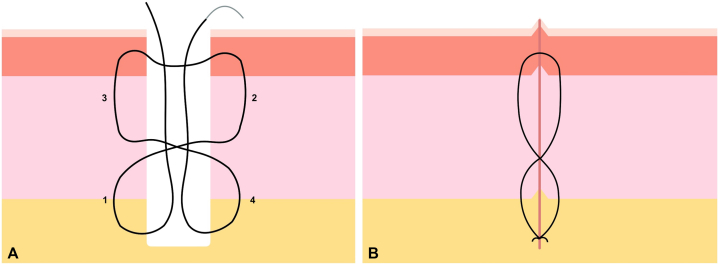
**A**, “Vertical-X–shaped stitch” guide. **B**, Finished suture guaranteeing the absence of dead space owing to the X-crossing of the sutures that provide extra anchorage at the central level.

However, in the case of a defect under tension, we normally use a variation of the subcutaneous or buried pulley stitch^2^ at 2 levels that we named stacked pulley stitch, which allows us to take advantage of the closing capacity of the pulley stitch and bring together the walls of the defect at different levels (Fig 2, *A*). The stitch also begins in the lower part in subcutaneous cellular tissue, with or without including deeper tissues, ascends, and exits through superficial subcutaneous tissue or reticular dermis. It crosses to the contralateral side, enters at the same level and descends again to deep subcutaneous tissue where it exits, thus completing the first loop of the pulley. Subsequently, it ascends through the center of the defect until it reaches the superficial subcutaneous tissue or reticular dermis of the contralateral side through which it enters and ascends until it exits through the papillary dermis. It crosses and enters at the same level of the other wall, descends, and exits through superficial subcutaneous tissue or reticular dermis to the center of the defect, completing the second loop, and then, it is knotted with the other end. As seen in the previous case, we achieve an optimal tissue approximation at all levels (Fig 2, *B*), reducing virtual spaces, and we take the advantage of the usefulness of the pulley stitch to close defects with high tension.

**Fig 2:**
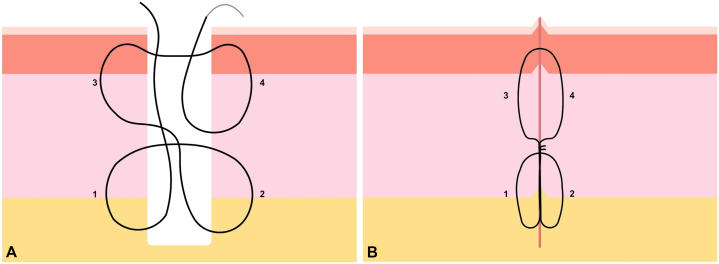
**A**, Four steps guide for “stacked pulley stich.” **B**, Completed suture showing the 2 pulley loops that facilitate the closure of defects with high tension and allow optimal defect closure at superficial, medium, and deep levels.

## Conflicts of interest

None disclosed.
